# Transcriptomic profiling reveals response mechanisms of *Lactuca indica* seedlings to seawater irrigation stress

**DOI:** 10.3389/fpls.2025.1599564

**Published:** 2025-06-03

**Authors:** Dingding Cao, Lirong Xiang, Ziling Li, Neng Wei, Qingfeng Wang

**Affiliations:** ^1^ Fujian Key Laboratory on Conservation and Sustainable Utilization of Marine Biodiversity, Fuzhou Institute of Oceanography, College of Geography and Oceanography, Minjiang University, Fuzhou, China; ^2^ College of Life Sciences, Hubei University, Wuhan, China; ^3^ State Key Laboratory of Plant Diversity and Specialty Crops, Wuhan Botanical Garden, Chinese Academy of Sciences, Wuhan, China; ^4^ Sino-Africa Joint Research Center, Chinese Academy of Sciences, Wuhan, China

**Keywords:** differential gene expression, salt tolerance, seawater irrigation, transcriptome analysis, wild vegetables

## Abstract

**Introduction:**

The increasing global soil salinization has accelerated research on seawater irrigation agriculture. Developing wild vegetables through seawater irrigation could establish foundational strategies for utilizing island vegetable germplasm resources.

**Methods:**

This study investigated two distinct leaf-shaped individuals (S and Y) of wild *Lactuca indica* (*L. indica*) through hydroponic experiments with diluted seawater during seedling stage. Physiological and morphological assessments revealed that Y exhibited superior seawater tolerance compared to S. Tissue-specific -plant transcriptome analysis identified key metabolic pathways and regulatory genes in roots, stems, and leaves.

**Results:**

Differential gene expression analysis showed tissue-specific enrichment patterns: leaves predominantly enriched light-harvesting complex (LHC) genes in photosynthesis pathways; stems exhibited upregulation in cutin, suberin, and wax biosynthesis pathways; while roots showed activation of nitrogen metabolism pathways.

**Discussion:**

Based on the data from transcriptomics, we infered that the key salt-tolerant candidate genes include: (1) leaf-specific LHC genes enhancing photosynthetic efficiency; (2) stem-expressed wax biosynthesis gene aldehyde decarbonylase CER1, and cytochrome P450 family members fatty acid omega-hydroxylase CYP86A4S and cytochrome P450 family 77 subfamily A (CYP77A); and (3) root-specific nitrogen metabolism regulators (nitrate reductase (NR), nitrate/nitrite transporter 2 (NRT2), and nitrite reductase (NirA). This study provides the comprehensive tissue-specific transcriptome profile of wild *L. indica* under seawater irrigation, predicting crucial metabolic pathways and candidate genes that might enhance seawater tolerance. Our findings establish a valuable reference for salt tolerance research in wild vegetables and offer potential genetic targets for improving crop resilience in saline-affected ecosystems.

## 1 Introduction

Global warming, rising sea levels, rapid industrialization and urbanization, and the escalating scarcity of freshwater resources have emerged as critical challenges (Bressan et al., 2009). In response, seawater irrigation agriculture has been developed. This practice involves irrigating salt‐tolerant crops with seawater, a mixture of seawater and freshwater, or saline groundwater, primarily in coastal tidal flats or in select inland saline–alkaline areas (Martínez-Alvarez et al., 2016). Notably, seawater irrigation agriculture accounts for approximately 20% of the world’s arable land and contributes about 40% to global grain production (Martinez-Alvarez et al., 2020; Martínez-Alvarez et al., 2018). Moreover, long-term seawater irrigation has been found to elevate soil pH, thereby mitigating soil acidification (Liang et al., 2023). In China, the abundant underground saline water resources present considerable potential for irrigation using slightly saline water (Yuan et al., 2019). Additionally, hydroponic cultivation in organic vegetable production offers significant advantages, as water-cultivated plants exhibit superior growth indices and higher yields compared to those grown in soil (Park and Williams, 2024). Research on vegetable seawater irrigation is of great practical value. For example, Gu et al. (2020) demonstrated that the inhibitory effects of seawater irrigation on celery growth intensified with increasing seawater concentration, with a more pronounced impact on the fresh weight of the aboveground parts than on the roots. In another study, Jiang (2017) evaluated the seawater irrigation cultivation of five wild vegetables—*Talinum paniculatum*, *Piper sarmentosum*, *Polygonum chinense*, *Anredera cordifolia*, and *Tetragonia tetragonioides*—using hydroponic experiments with various substrates and seawater concentrations. The results revealed a salt tolerance ranking of *Tetragonia tetragonioides* > *Talinum paniculatum* > *Piper sarmentosum* > *Polygonum chinense* > *Anredera cordifolia*, thus providing a theoretical basis for the introduction and cultivation of wild island vegetables. Furthermore, Li (2018) investigated the intraspecific variation in salt tolerance among F_2_ hybrids of *Agropyron cristatum* L. and *Agropyron mongolicum* Keng, highlighting differences in salt tolerance within the same species.

Salt stress can impede photosynthesis and inhibit cell division and expansion (van Zelm et al., 2020). Studies have demonstrated that light signals—such as light intensity, light quality, and photoperiod-play a critical role in modulating plant responses to salt stress (Assaha et al., 2017; Peng et al., 2025). In particular, high expression of the key photosynthetic gene encoding the LHC enhances plant salt tolerance, whereas its reduced expression compromises this tolerance (Chen et al., 2023; Jiang et al., 2014; Zhang et al., 2025). The epidermal cuticle of plants is also essential for adaptation to various abiotic stresses (Zhao et al., 2025). Under salt treatment, genes involved in cutin and wax biosynthesis-namely CYP86A4, CER1, CER2, and CER3-are significantly upregulated (Sun et al., 2024). Concurrently, salt stress inhibits the nutrient uptake capacity of plants, leading to nutrient imbalances, deficiencies, and disruptions in nutrient metabolism due to salt accumulation (Miura, 2013). Consequently, further investigation into the key regulatory pathways and genes associated with plant responses to salt stress is warranted.

*L. indica*, a wild edible green leafy vegetable rich in flavonoids and other secondary metabolites, is widely used in Asia as an antimicrobial and anti-inflammatory remedy. Its unique biochemical profile offers considerable potential for the development and application of functional foods (Chen et al., 2007; Hao et al., 2023; Kim et al., 2008; Lüthje et al., 2011).

In this study, we investigated the salt tolerance of seedlings of wild *L. indica* with two distinct leaf morphologies under seawater irrigation conditions. Through transcriptomic analysis, the underlying salt tolerance mechanisms were predicted, thereby establishing a theoretical foundation for the development of seawater irrigation strategies for wild vegetables.

## 2 Materials and methods

### 2.1 Plant materials

The plant materials used in this study were wild *L. indica* var. *laciniata*, preserved in the Marine Germplasm Resource Bank of the School of Geography and Oceanography, Minjiang University. Fully developed seeds were selected for germination experiments. The seeds were germinated in darkness for one week in an incubator set at 25°C before being subjected to seawater irrigation treatment.

The seedlings were cultivated under controlled conditions with a light intensity of 400 μmol/m²/s, a temperature of 25°C, and a photoperiod of 16 hours light/8 hours dark. The seawater used for irrigation was collected from the coastal area of Fuzhou, Fujian Province, China. It was diluted to one-sixth of its original concentration (seawater: freshwater = 1:6, v/v) for hydroponic seedling experiments. The reason why we chose this concentration is that our experiments found that lettuce seedlings could not survive after being irrigated with seawater at higher concentrations (such as 1:2 and 1:3 seawater concentrations).

First, measure the blade length (L) and width (W) of S and Y with a ruler, and then calculate the ratio (L:W) using the leaf L/W (Zhang et al., 2017). The measurement of the leaves contains five biological replicates.

### 2.2 Phylogenetic analysis

To determine the phylogenetic placement of *L. indica* **var.** *laciniata* individuals S and Y, we selected 86 ITS sequences from genus *Lactuca* as the ingroup and five species as the outgroup (*Prenanthes purpurea, Leontodon tuberosus, Hypochaeris radicata, Soroseris erysimoides*, and *Nabalus tatarinowii*). PCR amplification, sequencing, and data analysis were conducted following the method described by Kocyan et al. (2007).

In this study, two sequences (S and Y) were newly amplified, while all other sequences were retrieved from GenBank (**Table 1**). Genomic DNA of S and Y was extracted using a modified CTAB method (Allen et al., 2006). The ITS (internal transcribed spacer) region was selected for analysis, and sequencing primers were adopted from White’s study (White et al., 1990). ITS sequences were aligned using the MUSCLE algorithm, followed by manual adjustments in Geneious Pro 5.6.4 (Kearse et al., 2012). ModelFinder (Kalyaanamoorthy et al., 2017) was used to select the best-fit partition model (Edge-unlinked) under the Bayesian information criterion. The phylogenetic tree was reconstructed using maximum likelihood (ML) analysis in IQ-TREE v. 1.6.8 (Nguyen et al., 2015). For detailed methods in phylogenetic tree reconstruction, refer to Wei et al. (2020). The final phylogenetic tree was shown using the iTOL web server (Letunic and Bork, 2016).

**Table 1:** Leaf morphology statistics of the two wild *L. indica* var. *laciniata* individuals.

Name	Length (cm)	Width (cm)	L:W
S	15.52 ± 1.21	1.80 ± 0.15	8.65 ± 0.66^b^
Y	20.47 ± 1.43	4.53 ± 1.44	4.53 ± 0.61^a^

The letters a and b above the numbers indicate the significant differences between S and Y.

### 2.3 Catalase activity assay

CAT activity was determined using the method described by Sun et al. (1988). The reaction was rapidly terminated by adding ammonium molybdate, which forms a pale yellow complex with residual H_2_O_2_. The reaction time was set to 60 seconds. One unit (U) of CAT activity was defined as the amount of enzyme required to decompose 1 µmol of H_2_O_2_ per second per milligram of protein.

### 2.4 Superoxide dismutase activity assay

SOD activity was measured according to the method of Sun et al. (1988). The assay is based on the reduction of nitroblue tetrazolium (NBT) by superoxide radicals, resulting in the formation of a blue formazan product. The absorbance of the blue product was measured at 560 nm as an indicator of SOD activity.

### 2.5 Transcriptome sequencing and analysis

#### 2.5.1 RNA extraction

Seedlings of S and Y subjected to seawater irrigation treatment were collected, and transcriptome analysis was performed on leaf, stem, and root samples. Each analysis was conducted in triplicate. Total RNA of leaf of S (SL), stem of S (ST), root of S (SR), leaf of Y (YL), stem of Y (YS), and root of Y (YR) was extracted using the Tiangen RNA Extraction Kit (Tiangen, China). High-quality RNA samples were used for transcriptome library construction. We evaluated the RNA quality by RNA mass number (RQN), where the RQN of SL, ST, SR, YL, YS, and YR were 8.6, 9.9, 10, 8.7, 10, and 10, respectively. The RQN value detection was accomplished through Agilent 5300. For transcriptome sampling, we selected three uniformly growing seedlings for mixed sampling. That is, the leaves, roots, and stem segments of the three seedlings were mixed respectively to extract RNA (a total of six RNA samples, namely SL, ST, SR, YL, YS, and YR, and then the RNA of each sample was divided into three parts and sequenced in sequence on the machine (that is, there were a total of 18 transcriptome sequencing samples). They were respectively SL_1, SL_2, SL_3, ST_1, ST_2, ST_3, SR_1, SR_2, SR_3, SL_1, SL_2, YL_3, YS_1, YS_2, YS_3, YR_1, YR_2, and YR_3.

#### 2.5.2 Library preparation and sequencing

RNA purification, reverse transcription, library construction and sequencing were performed at Shanghai Majorbio Bio-pharm Biotechnology Co., Ltd. (Shanghai, China) according to the manufacturer’s instructions. The lettuce RNA-seq transcriptome library was prepared following Illumina^®^ Stranded mRNA Prep, Ligation (San Diego, CA) using 1μg of total RNA. Shortly, messenger RNA was isolated according to poly A selection method by oligo (dT) beads and then fragmented by fragmentation buffer firstly. Secondly double-stranded cDNA was synthesized with random hexamer primers. Then the synthesized cDNA was subjected to end-repair, phosphorylation and adapter addition according to library construction protocol. Libraries were size selected for cDNA target fragments of 300-400bp use magnetic beads followed by PCR amplified for 10–15 PCR cycles. After quantified by Qubit 4.0, the sequencing library was performed on NovaSeq Xplus platform (PE150) using NovaSeq Reagent Kit.

#### 2.5.3 Quality control and *de novo* assembly

The raw paired end reads were trimmed and quality controlled by fastp (Chen et al., 2018) with default parameters. Then cleandata from the samples were used to do *de-novo* assembly with Trinity (Grabherr et al., 2011). To increase the assembly quality, all the assembled sequences were filtered by CD-HIT (Fu et al., 2012) and TransRate (Smith-Unna et al., 2016) and assessed with BUSCO (Benchmarking Universal Single-Copy Orthologs) (Manni et al., 2021). The assembled transcripts were searched against the NCBI protein nonredundant (NR), Clusters of Orthologous Groups of proteins (COG), and Kyoto Encyclopedia of Genes and Genomes (KEGG) (Ogata et al., 1999) databases using Diamond to identify the proteins that had the highest sequence similarity with the given transcripts to retrieve their function annotations and a typical cut-off E-values less than 1.0x10–^5^ was setBLAST2GO (Conesa et al., 2005) program was used to get GO annotations of unique assembled transcripts for describing biological processes, molecular functions and cellular components.

#### 2.5.4 Differential expression analysis and functional enrichment

To identify DEGs (differential expression genes) between two different samples/groups, the expression level of each transcript was calculated according to the transcripts per million reads (TPM) method. RSEM (Li and Dewey, 2011) was used to quantify gene abundances. Essentially, differential expression analysis was performed using the DESeq2 (Love et al., 2014; Vaidya et al., 2011). Genes with the expression of |log2FC|≥1 and FDR< 0.05 (DESeq2) were considered to be DEGs.

Expression level difference analysis is a statistical inference process for determining whether differentially expressed genes/transcripts have occurred in all detected genes/transcripts. Due to the large number of involved genes/transcripts, multiple statistical tests (the number of tests is the number of genes to be tested) are required. To control the probability or frequency of errors in the overall inference results, the p-value obtained from statistical tests is corrected, that is, multiple test correction is carried out. The corrected p-value is called Padjust. The method of multiple test correction in this study is BH (False Discovery Rate Correction with Benjamin ini/Hochberg) (Haynes, 2013). The analysis and plotting of the DEGs function-enriched part were carried out through the Majorbio Cloud platform (Han et al., 2024).

### 2.6 qRT-PCR

qRT-PCR was performed in CFX Connect (BIO-RAD) using the SYBR Green Master Mix, and amplified with 1 μL of cDNA, 5 μL of 2 × SYBR Green Master Mix, and 0.6 μM of each primer. The amplification program is consisted of 1 cycle of 95°C for 15 minsminutes, and 40 cycles of 95°C for 15 s and 58°C for 30 s. The relative expression of target genes was normalized by comparison with the reference β-actin (TRINITY_DN8930_c1_g2) and analyzed using the 2^-ΔΔCT^ Method (Livak and Schmittgen, 2001). The primers used were listed in **Supplementary Table S6**.

### 2.7 Statistical analysis

Statistical analyses were performed using SPSS 21.0 (IBM Corp., Armonk, NY, USA). When conducting significance analysis and correlation analysis on all data, the test of normality of variance was performed first. The detection method for the normal distribution of data is to draw the histogram of the data and determine whether the data conforms to the normal distribution through the Shapiro-Wilk Sig value test (if Sig>0.05, the data is normally distributed; otherwise, it is not normally distributed). For the data of significant difference analysis, the homogeneity of variance test was conducted. The method was the Levene test. For the comparison of significant differences between the two groups of data that conformed to the normal distribution, the independent sample t-test was selected. For the comparison of significant differences between the two groups of data that did not conformed to the normal distribution, the Mann-Whitney U test among the non-parametric tests was chosen. For the comparison of significant differences among multiple groups of data, the Duncan s multiple range test was selected. When the P value of the analysis result is less than 0.05, it indicates a significant difference. The results of the correlation analysis were expressed by the Spearman coefficient. All data are presented as mean ± standard error (SE).

## 3 Results

### 3.1 Two distinct leaf morphologies in *L. indica* var. *laciniata* individuals S and Y

**Figure 1:**
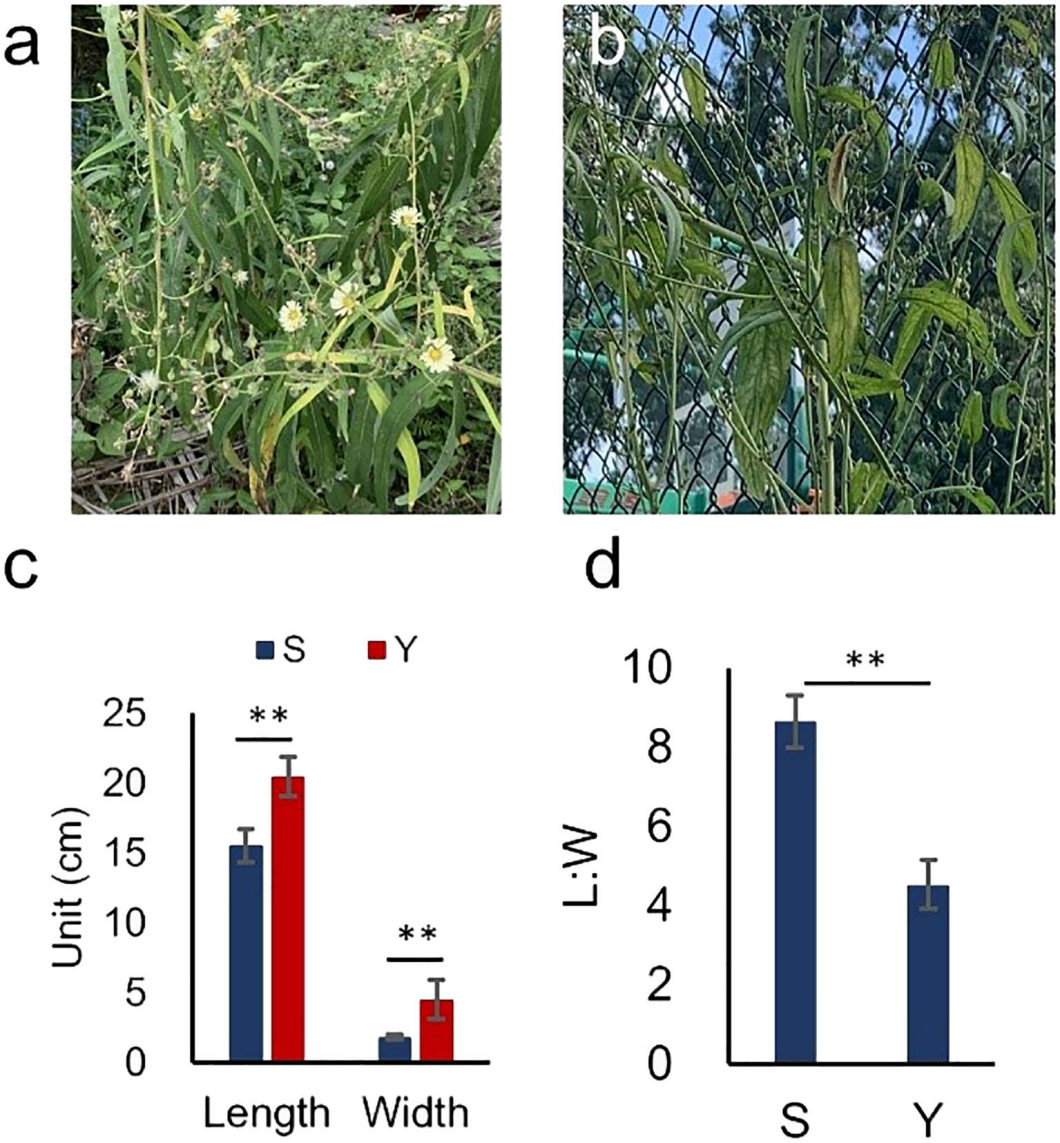
Two Individuals of *L. indica.* **(a, b)** Representative images of S and Y. **(c, d)** Statistical analysis of leaf length, width, and L:W for S and Y. Asterisks indicate significant differences (*P*< 0.01).

In the germplasm resource bank, we identified two individuals of *L. indica* var. *laciniata* with distinct leaf morphologies. Upon cultivation, we observed notable differences in leaf shape between them: one with relatively narrow leaves (referred to as S) and the other with broader leaves (referred to as Y) (**Figures 1a, b**).

The leaf length-to-width ratio (L:W) was 8.65 for S and 4.53 for Y, with S exhibiting a significantly higher L:W compared to Y (**Table 1**, **Figures 1c, d**). Morphologically, S had a narrow, elongated leaf shape, whereas Y displayed a more oblong leaf form.

**Figure 2:**
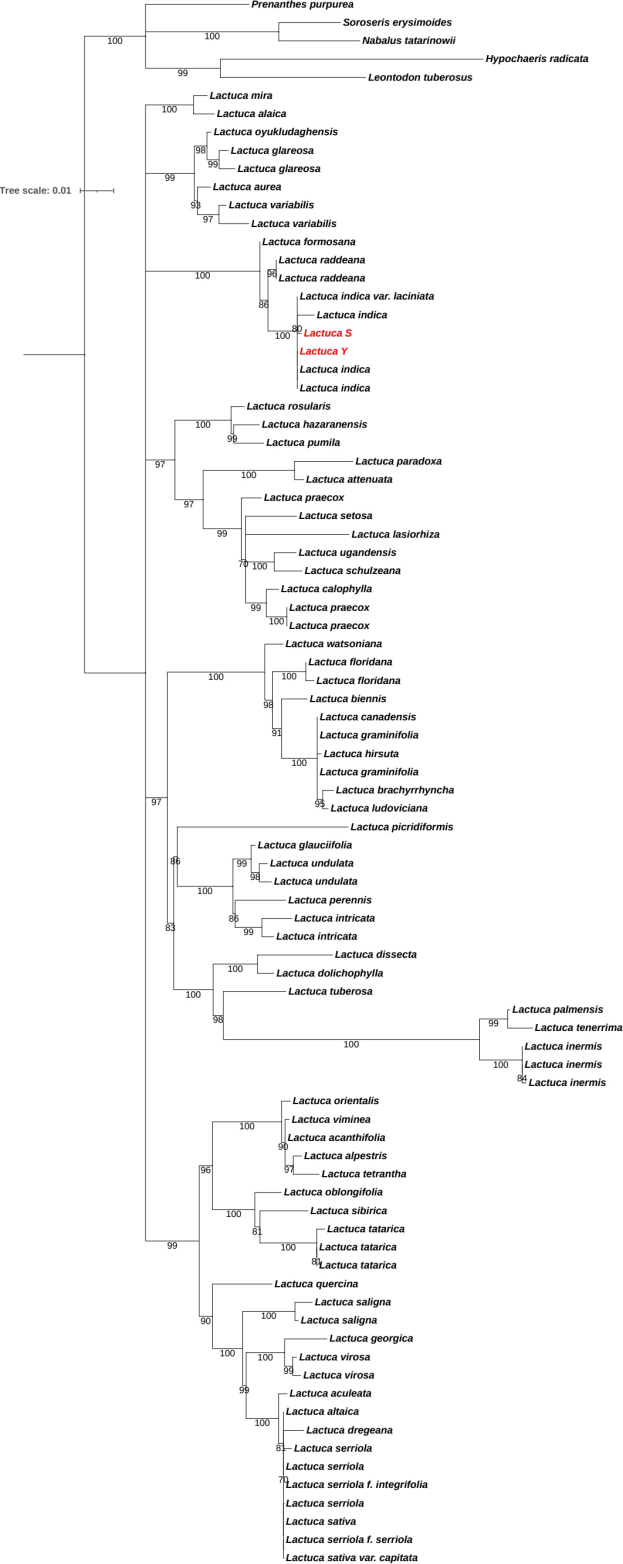
Molecular Phylogenetic tree. Maximum likelihood tree inferred from the ITS dataset of *Lactuca* and outgroup species to elucidate the phylogenetic position of S and Y, which are highlighted by red. The nodes with bootstrap value below 70 are collapsed.

We conducted molecular identification of the S and Y with distinct leaf morphologies. Maximum likelihood tree was inferred from the ITS dataset of *Lactuca* and outgroup species to elucidate the phylogenetic position of S and Y, shown in **Figure 2**. Both S and Y clustered within *L. indica* with a bootstrap support value of 100, confirming that both S and Ybelong to *L. indica* var. *laciniata*.

### 3.2 Effects of seawater irrigation on seedlings of two wild *L. indica* var. *laciniata* individuals

**Figure 3:**
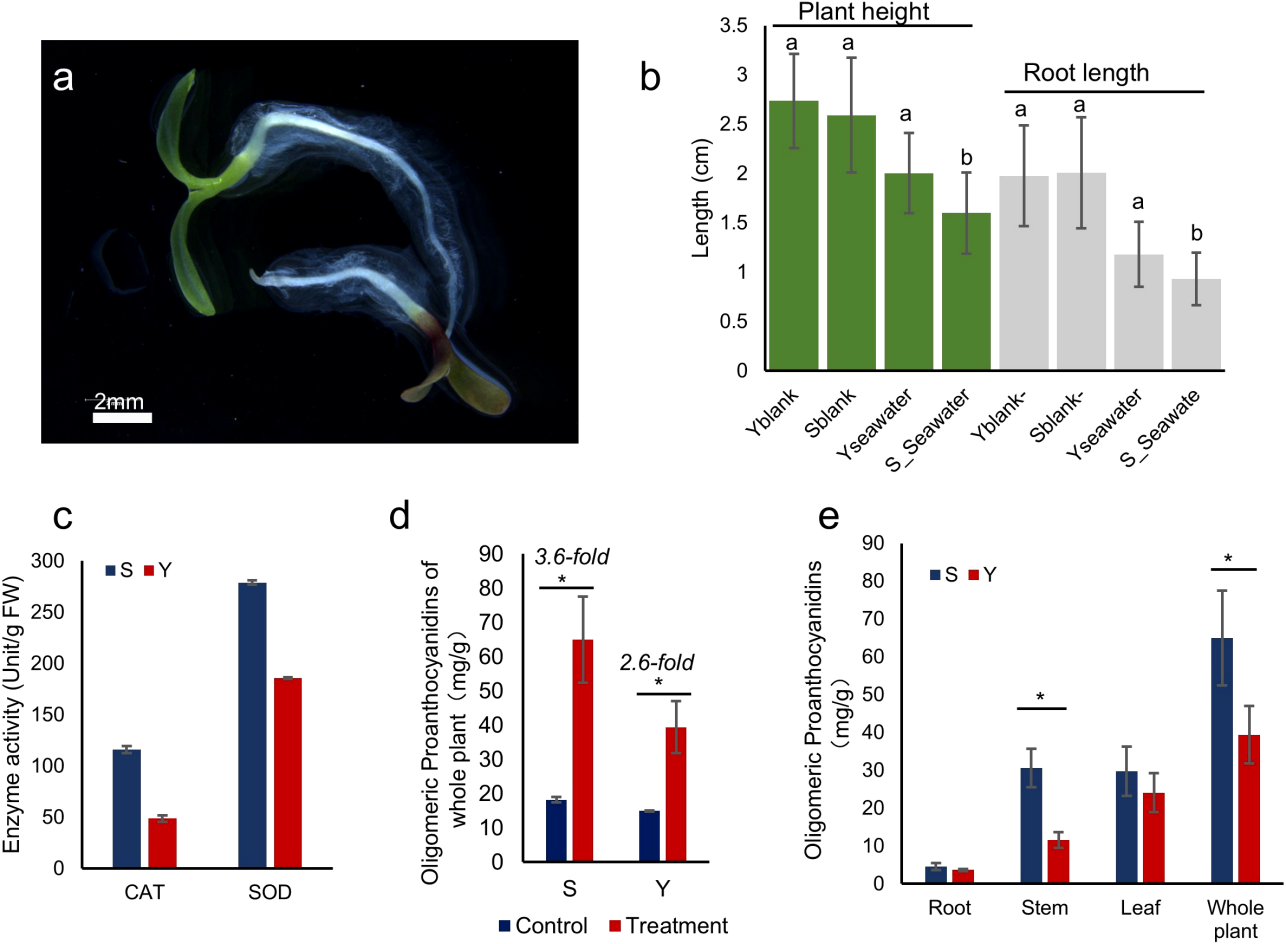
Germination of S and Y Under Seawater Conditions. **(a)** Seedlings of S and Y germinated in one-sixth diluted seawater hydroponic conditions (Y at the top, S at the bottom). **(b)** Plant height and root length of germinated seedlings. The letters a and b above the bar chart represent the significant differences of the Seawater treatment group compared with the blank. **(c)** Total proanthocyanidin content in whole plants of *L. indica* var. *laciniata* under one-sixth seawater treatment (Treatment) and control (Control) conditions. **(d)** Proanthocyanidin content in different plant tissues of germinated seedlings. **(e)** Antioxidant enzyme activity in germinated seedlings. *indicates significant differences (*P*< 0.05).

A one-week seawater irrigation experiment was conducted on seedlings of S and Y using one-sixth diluted seawater (**Figure 3a**). After one week, we analyzed their growth and physiological parameters. Compared to freshwater germination, seawater irrigation significantly inhibited plant height and root length in S, whereas Y showed no significant reduction in these growth parameters (**Figure 3b**).

To assess oxidative stress levels, we measured the activity of reactive oxygen species (ROS)-related enzymes in the leaves of seawater-treated plants. The activities of catalase (CAT) and superoxide dismutase (SOD) were higher in S compared to Y, indicating that S at the seedling stage was more sensitive to seawater irrigation (**Figure 3c**).

Furthermore, we quantified proanthocyanidin content in different plant tissues under seawater treatment. The total proanthocyanidin content in both S and Y increased significantly, with S showing a 3.6-fold increase and Y showing a 2.6-fold increase (**Figure 3d**). S accumulated more proanthocyanidins than Y across all tissues, with leaf proanthocyanidin levels 1.25 times higher, root levels 1.44 times higher, and stem levels 1.23 times higher than those in Y. The most pronounced difference in proanthocyanidin accumulation between them was observed in the stems (**Figure 3e**).

### 3.3 Transcriptional regulatory mechanisms of two wild *L. indica* individual under seawater irrigation stress

The wild *L. indica* S and Y, which exhibit typical leaf morphology differences, respond to seawater irrigation stress by accumulating varying levels of anthocyanins and antioxidant enzymes. This study focuses on these fascinating phenomena and explores the transcriptional responses and regulatory mechanisms of S and Y under seawater irrigation stress. We conducted transcriptomic sequencing on the roots, stems, and leaves of both varieties after seawater irrigation treatment.

**Figure 4:**
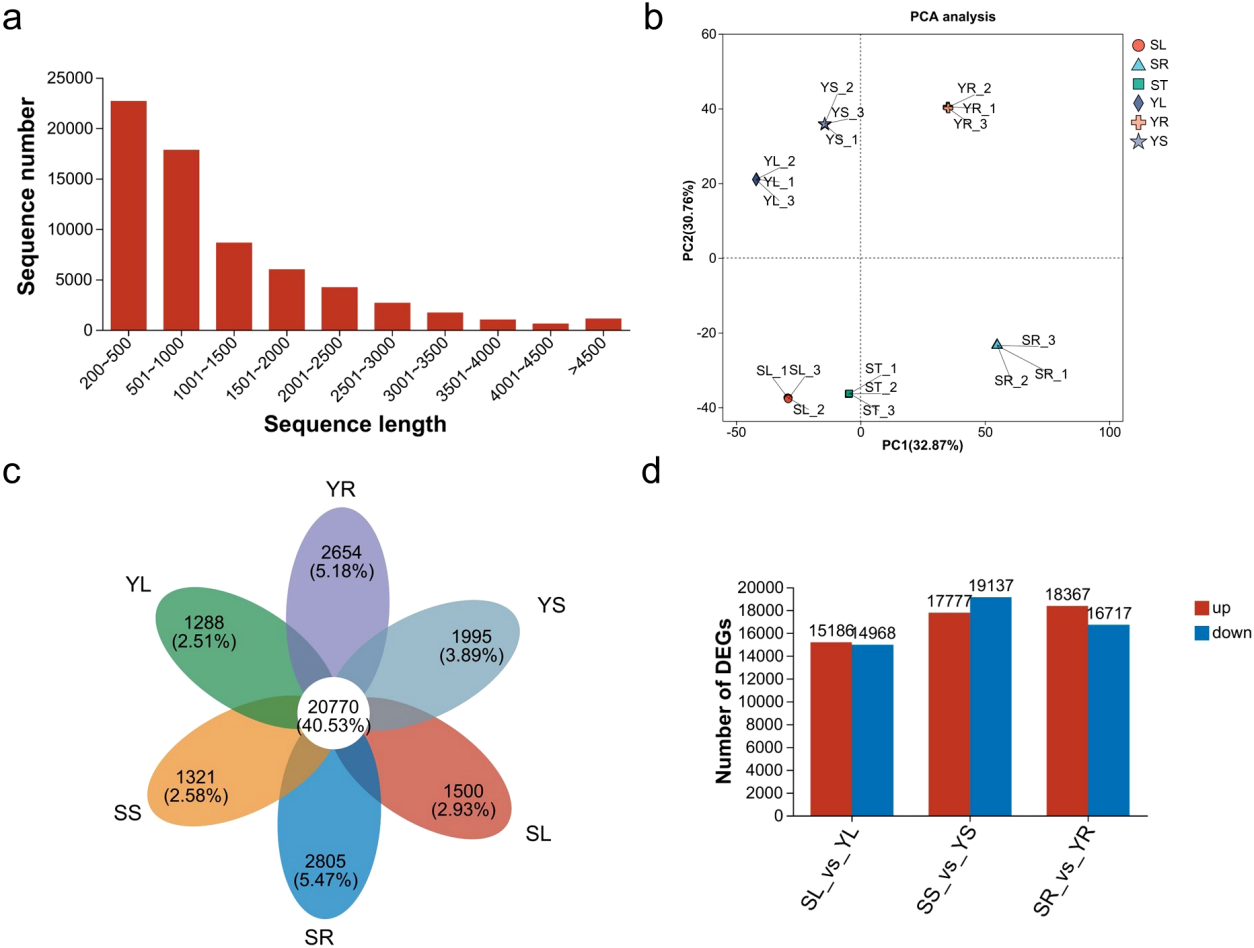
Overview of transcriptomic analysis results. **(a)** Sequence length distribution. **(b)** PCA analysis of S and Y samples. **(c)** Venn diagram of unigenes in different transcriptome datasets. **(d)** Bar chart of upregulated and downregulated unigenes in the leaves, stems, and roots of S *vs*. Y.

A total of six transcriptome datasets were generated (ST, SR, SL, YL, YR, YS), comprising 130,366 transcripts, with 66,621 unique genes identified. The transcript N50 length was 2,073 bp, the fragment mapping percentage was 93.221%, and the BUSCO score was C: 93.7% [S: 57.2%; D: 36.5%], indicating high-quality sequencing and assembly. The sequencing length distribution (**Figure 4a**) was primarily concentrated between 200 and 1,500 bp, accounting for 74% of the total sequences. We explored the relationships and variations among the root, stem and leaf samples of S and Y through principal component analysis (PCA). The results showed the separation of S (SL, ST, and SR) and Y (YL, YR, and YS) (**Figure 4b**). Additionally, the number of genes in each group is shown in **Figure 4c**. A comparative analysis of the transcripts across different tissues of S and Y is presented (**Figure 4d**, **Supplementary Table S1-4**).

### 3.4 Differentially expressed gene GO and KEGG enrichment analysis of S and Y

Based on the physiological data from seawater irrigation experiments, which showed that Y exhibited higher salt tolerance than S, we focused on genes with high expression in various tissues of Y. We conducted a visual analysis of the expression distribution of DEGs (**Supplementary Figure S1**). The upregulated genes in the stem, root, and leaf tissues of Y were 9,064, 7,343, and 6,305, respectively. To double-check the reliability of the transcriptome data, we selected some genes for qRT-PCR experiments and jointly analyzed the results with the transcriptome expression levels. The results also confirmed that our transcriptome data were stable and reliable (**Supplementary Figure S2**).

**Figure 5:**
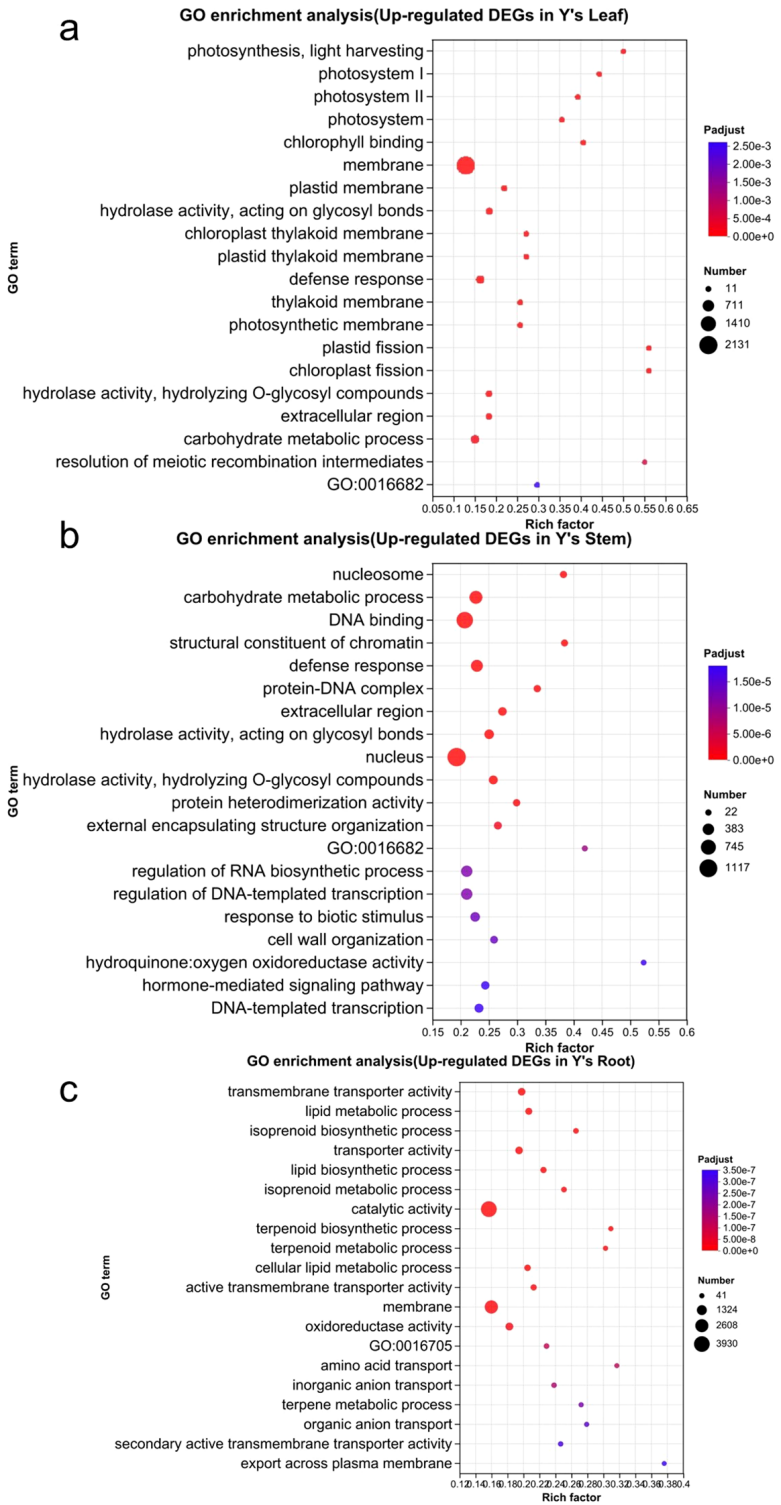
The GO enrichment analysis of upregulated genes in Y. The GO enrichment analysis of upregulated genes in the leaves, stems, and roots of Y is shown in panels a-c. GO:16682, oxidoreductase activity, acting on diphenols and related substances as donors, oxygen as acceptor;GO:16705, oxidoreductase activity, acting on paired donors, with incorporation or reduction of molecular oxygen.

We then performed GO and KEGG functional annotation and enrichment analysis of the upregulated genes in Y *vs*. S (**Figure 5**). The GO enrichment analysis revealed that upregulated genes in the leaf of Y *vs*. S were mainly enriched in the following terms: membrane (2,131), carbohydrate metabolic process (334), defense response (293), photosystem I, photosystem II, and photosystem (95), and photosynthesis, light harvesting (29). In the stem, the upregulated genes were primarily enriched in nucleus (1,117), DNA binding (879), carbohydrate metabolic process (506), and nucleosome (89). In the root, upregulated genes were mainly enriched in catalytic activity (3,930), membrane (2,663), oxidoreductase activity (675), transmembrane transporter activity (608), and transporter activity (615).

**Figure 6:**
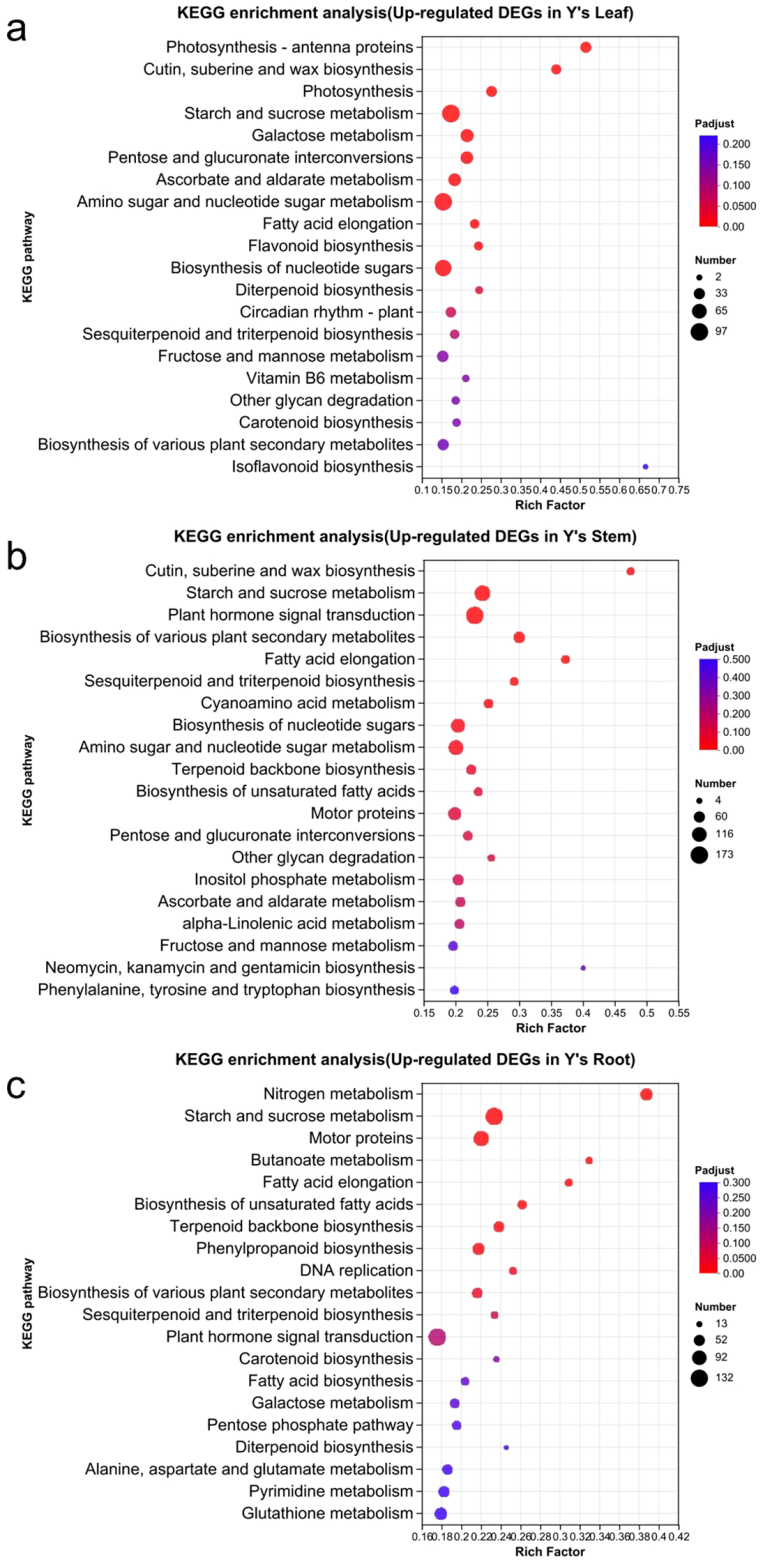
KEGG enrichment analysis of upregulated genes in Y. KEGG enrichment analysis of upregulated genes in Y, including the analysis of GO enrichment in the leaves, stems, and roots of Y **(a-c)**.

KEGG enrichment analysis revealed that in the upregulated genes of Y *vs*. S, the leaf was primarily enriched in the following pathways (**Figure 6**): Starch and sucrose metabolism (97), Amino sugar and nucleotide sugar metabolism (94), Biosynthesis of nucleotide sugars (82), Photosynthesis and Photosynthesis-antenna protein (64), and Cutin, suberine, and wax biosynthesis (26). In the stem, the upregulated genes were mainly enriched in Plant hormone signal transduction (173), Starch and sucrose metabolism (135), Amino sugar and nucleotide sugar metabolism (122), Biosynthesis of various plant secondary metabolites (68), and Cutin, suberine, and wax biosynthesis (28). In the root, the upregulated genes were primarily enriched in Starch and sucrose metabolism (130), Motor proteins (102), and Nitrogen metabolism (67).

**Figure 7:**
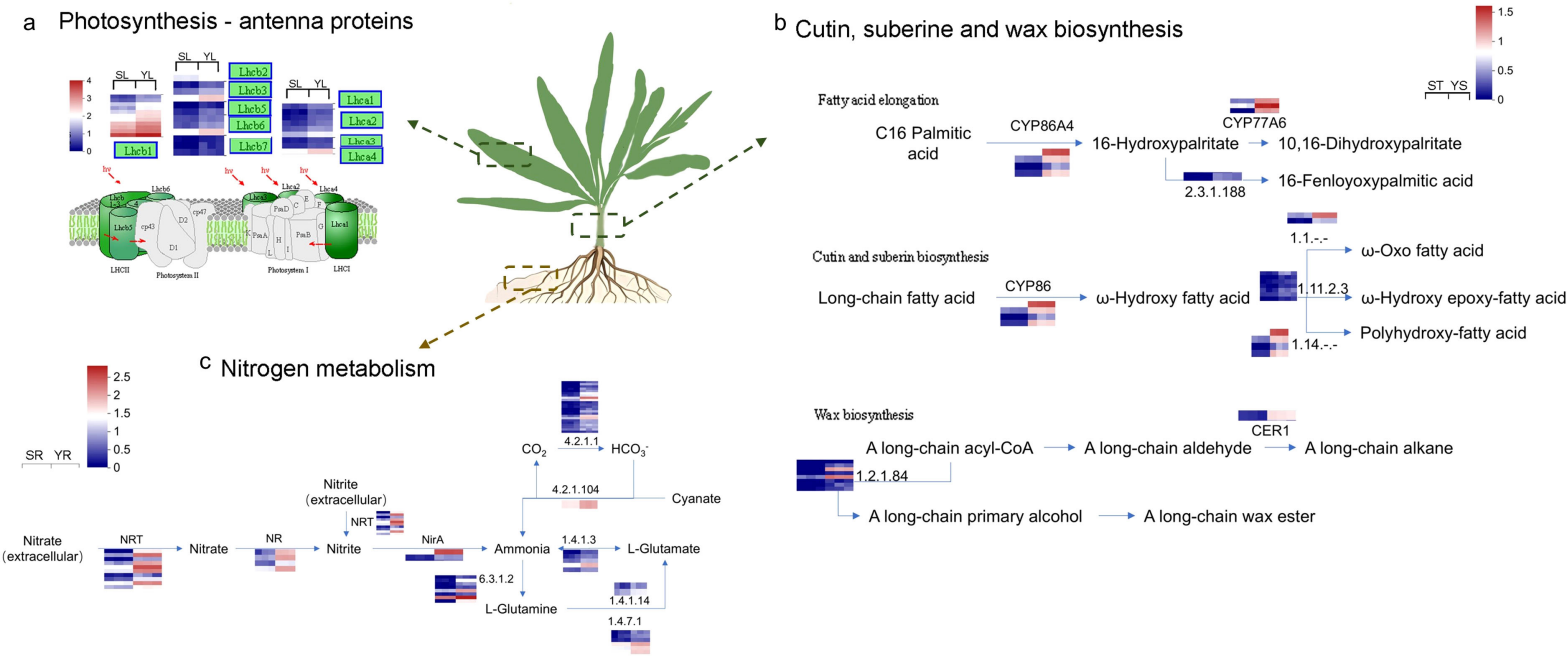
Key Pathways Responding to Salt Stress in Different Parts of *L. indica* Y. **(a)** Photosynthesis and metabolic pathways in the leaves of *L. indica*; **(b)** Cutin, suberine, and wax biosynthesis in the stem of *L. indica*; **(c)** Nitrogen metabolism in the roots of *L. indica*. In the figure, blue indicates upregulated genes in Y *vs*. S, while red represents downregulated genes in Y *vs*. S. The heat map near the gene in the figure showed the relative expression levels of the gene and its transcript in the transcriptome in S and Y. For example, for the Lhcb1 gene in figure a, the horizontal coordinates of the heat map were SL_1, SL_2, SL_3, YL_1, YL_2, and YL_3 respectively.

Through KEGG enrichment analysis of upregulated genes in Y, we identified key pathways of interest in the root, stem, and leaf tissues (**Figure 7**). In the leaf, upregulated expression was primarily associated with genes related to the LHC in the Photosynthesis pathway, especially with a significant upregulation of Lhcb-related genes in Photosystem I. In the root, the upregulated DEGs were enriched in the Nitrogen metabolism pathway, which focused on the intracellular conversion of Nitrate to Ammonia through the NR and NirA genes, as well as the extracellular conversion of Nitrate to Ammonia via Nrt, which further flows into Glutamate metabolism. In the stem, the Cutin, suberine, and wax biosynthesis pathway mainly involved fatty acid elongation. The upregulated expression of members of the P450 gene family, including CYP86, CYP70, and CYP77, promoted carbon chain elongation, resulting in the formation of long-chain fatty acids such as 22-Hydroxydocosanoate, Polyhydroxy-fatty acid, ω-Oxo fatty acid, ω-Hydroxy epoxy-fatty acid, and Polyhydroxy-fatty acid. Additionally, interestingly, in the wax biosynthesis pathway, the upregulation of genes like CER1 promoted the synthesis of long-chain wax esters.

This study focuses on the seedling growth experiment of lettuce under seawater irrigation conditions. Through double check of transcriptomics and qRT-PCR, we identified several candidate genes that might promote the salt tolerance of lettuce seedlings, including LHCB1-3/5-7, LHCA1-4, NR, NRT2, NirA, CYP86A4S, CYP77A and CER1. Overall, through transcriptomic analysis, we hypothesize the regulatory mechanisms underlying the salt tolerance of Y under seawater irrigation. Compared with the narrow leaf shape of S, the broader leaf morphology of Y seems to offer a larger leaf area, which may be beneficial for Y to provide energy through photosynthesis to cope with salt stress, which is the focus of our subsequent work. Additionally, Y’s stem might be capable of synthesizing more waxes than S, serving a protective function. The root system may enhance nitrogen metabolism, boosting the plant’s nutrient assimilation.

## 4 Discussion

### 4.1 Salt tolerance in *L. indica* seedlings

Under salt-induced oxidative stress, plants increase the activity of antioxidant enzymes to alleviate the damage caused by stress, and the activity of key enzymes in the ROS scavenging system reflects the plant’s tolerance to salt stress (Li et al., 2004). Research on *Lactuca sativa* indicates that NaCl-induced oxidative stress leads to lipid peroxidation and membrane damage, with increased activity of CAT and SOD (Eraslan et al., 2007). Studies on rice show that, compared to salt-tolerant varieties, salt-sensitive varieties exhibit a stronger induction of CAT and SOD activity under salt stress (Chawla et al., 2013). In the present study, after one week of treatment with seawater irrigation for seedlings, the activity of CAT and SOD in S were higher than those in Y, indicating that the antioxidant enzyme system of Y was more stable under seawater stress.

Proanthocyanidins are oligomers and polymer end products of the flavonoid biosynthesis pathway. The content of proanthocyanidins in plants is regulated by environmental signals (such as light stress) and genetic networks (Dixon et al., 2005). Proanthocyanidin pretreatment in cucumber seedlings enhances their resistance to high irradiation (HI), polyethylene glycol (PEG), and cold stress, suggesting a correlation between proanthocyanidins and stress tolerance (Zhu et al., 2017). Besides, proanthocyanidins have strong oxidizing properties and are beneficial to human health in terms of antioxidation, neuroprotection and antibacterial properties, so they are used as food additives (Rauf et al., 2019). This study speculated that S is more sensitive to salt stress caused by seawater irrigation, and thus accumulates more proanthocyanidins in the stems. This may provide an enrichment method for the subsequent extraction of proanthocyanidins in lettuce. Furthermore, the significant effect of seawater irrigation on increasing the proanthocyanidins content in lettuce S suggests its potential for proanthocyanidins purification and medicinal applications.

### 4.2 Analysis of salt tolerance mechanisms in *L. indica*

Salt stress is one of the major abiotic stresses that limit plant growth, affecting processes such as photosynthesis, protein synthesis, and lipid metabolism (Bagchi et al., 2000). Aboveground, salt stress is primarily characterized by reduced photosynthesis, while the underground parts, particularly the roots, experience inhibited water transport, which in turn affects nutrient absorption. In this study, transcriptomic analysis identified salt-tolerant candidate genes in *L. indica*, including LHC genes highly expressed in the leaves, wax biosynthesis-related genes CER1, cytochrome P450 family members CYP86A4S and CYP77A in the stem, and NR, NRT2, and NirA genes highly expressed in the roots.

Under seawater irrigation conditions, the high expression of photosystem-related genes in Y facilitated the efficient operation of photosynthesis, thus demonstrating higher salt tolerance in Y compared to S. In this study, a large number of light-harvesting complex genes were induced and upregulated in the leaves of Y under seawater irrigation, including LHCB1-3\5-7, and LHCA1-4. Light-harvesting complex gene expression can be induced by abiotic stresses. Studies on *Brassica campestris L.* (rapeseed) showed that overexpression of the *BcLhcb2.1* gene from rapeseed in *Arabidopsis thaliana* enhanced its resistance to cold, salt, and drought (Zhang et al., 2025). Similarly, in two cultivated species of celery (*Apium graveolens*), Lhcb1 expression was upregulated under heat, salt, and drought stress (Jiang et al., 2014).

The waxes in the plant cuticle play a protective role against abiotic stress (Lewandowska et al., 2020; Shepherd and Wynne Griffiths, 2006). Alkaline stress (NaHCO_3_) significantly alters the morphology of the epidermal wax in rice (Yang et al., 2015). In *Thellungiella salsuginea* (salt cress), salt-induced gene *TsnsLTP4* increased wax content in both the leaves and stems of *Arabidopsis thaliana*, thereby enhancing salt tolerance, suggesting that wax contributes to plant salt resistance (Sun et al., 2015). In cucumber, the CER1 gene regulates the biosynthesis of very long-chain (VLC) alkanes, with high expression in waxy fruit types. The expression of *CsCER1* is induced by cold, drought, salt stress, and abscisic acid, and it influences the cuticular properties and drought resistance of cucumber (Wang et al., 2015).

Additionally, cytochrome P450 (CYPs) is the largest enzyme family involved in NADPH- and/or O_2_-dependent hydroxylation reactions, playing a central role in the detoxification of exogenous substances. The expression of some CYP genes is regulated by environmental stresses, making them potential candidate genes for developing stress-resistant crop varieties (Pandian et al., 2020). The CYPs *SmCYP78A7a* promotes salt tolerance in eggplant through a positive feedback loop with *SmWRKY11* (Shen et al., 2024).

The high expression of NR, NRT2, and NirA in the roots promotes nitrogen metabolism under salt stress in *L. indica*. In a study on the salt tolerance of Tartary buckwheat (*Fagopyrum tataricum*), moderate low-concentration salt stress (2 g/kg) promoted root growth and enhanced the content of nitrogen metabolism-related substances and enzyme activities, while higher concentrations of salt stress (5 g/kg) inhibited nitrogen metabolism (Zhang et al., 2023). NR and NiR mediate the activation of inorganic nitrogen utilization (Liu et al., 2022). The expression of NR is closely related to salt tolerance in rice seedlings. A study on rice seedling salt tolerance found that seedlings grown under nitrate nitrogen ( $NO_{3}^{-}\;-\;N$ ) conditions exhibited stronger salt tolerance than those grown under ammonium ( $NH_{4}^{+}$ ) conditions. Under both normal and salt stress conditions, $NO_{3}^{-}$ significantly induced NR activity and nitric oxide (NO) production. Exogenous addition of the NO donor sodium nitroprusside (SNP) also enhanced seedling salt tolerance. The results suggest that NR-dependent NO production mediates the salt tolerance conferred by nitrate in rice seedlings (Teng et al., 2025). In *Suaeda salsa*, SsNRT2.1 can be induced by 200 mM NaCl, and overexpression of SsNRT2.1 in *Arabidopsis thaliana* increases the $NO_{3}^{-}$ content in the plant (Liu et al., 2021).

## 5 Conclusion

This study focused on two individuals of *L. indica* with distinct leaf shapes: the S individual with long, narrow leaves, and the Y individual with more oval-shaped leaves. They were confirmed through phylogenetic analysis based on ITS dataset. A seawater irrigation experiment was conducted, and the results showed that the Y exhibited higher salt tolerance than the S. By performing transcriptome sequencing on the roots, stems, and leaves of both S and Y, we predicted candidate genes associated with salt tolerance in *L. indica*. These include LHC genes highly expressed in the leaves, wax biosynthesis-related CER1 in the stems, cytochrome P450 family members CYP86A4S and CYP77A, and NR, NRT2, and NirA in the roots. This study systematically analyzed the possible synergy between the aboveground and underground parts of *L. indica* under seawater irrigation stress, providing insights into the transcriptional regulation mechanisms of salt stress response. These findings lay a theoretical foundation for future applications of seawater irrigation in agriculture.

## Data Availability

The transcriptomics data have been deposited to NCBI (https://www.ncbi.nlm.nih.gov/) with the accession numbers PRJNA1236924.

## Supplementary material

Supplementary Figure 1 Scatter plot of DEGs expression level difference statistics and MA plot. **(a-c)** Scatter plot of differentially expressed genes in different plant tissue parts of S and Y, for example, in SR *vs*. YR, the meaning of upregulated expression genes is that the expression level of the gene in SR is relatively higher than that in YR. A-c, the horizontal and vertical coordinates respectively represent SR and YR **(a)**. The expression levels of unigene (the longest transcript in the transcript cluster is regarded as unigene) in each of the three control groups SS and YS **(b)**, SL and YL **(c)**, with two samples in each group. Here, the values on the horizontal and vertical coordinates have been logarithmically processed, and each point represents a specific unigene. In the figure, the red dots represent significantly upregulated unigene, the blue dots represent significantly downregulated unigene, and the gray dots represent unigene with non-significant differences. After mapping all unigene onto it, the closer the point is to 0, the lower the expression level. Those points with a greater degree of deviation from the diagonal indicate that the expression difference of the unigene between the two samples is greater. **(d-f)** S and Y MA plots of differentially expressed genes in different plant tissue parts, d.SR *vs*. YR, e. SS *vs*. YS, and f. SL *vs*. YL. The horizontal axis represents the calculation based on the expression values of the difference control groups, and the vertical axis represents the expression changes of unigene between the difference control groups obtained from the difference expression analysis. The values of both the horizontal and vertical axes have been logarithmically processed. Each point in the figure represents a specific unigene. By default, red points indicate significantly up-regulated unigene, blue points indicate significantly down-regulated unigene, and gray points indicate non-significantly different unigene. After mapping all the unigene onto it, it can be known that the points at the top are the unigene with upregulated expression differences, and the points at the bottom are the unigene with downregulated expression differences. The points closer to the top and bottom have more significant expression differences. TPM, Transcripts Per Million reads.

Supplementary Figure 2 Analysis of qRT-PCR results and transcriptome expression levels and their correlations. **(a-h)** Gene expression in the stem. **(i-m)** Gene expression in the leaves. **(n-r)** Gene expression in the root. The header of the table represents the gene id. The blue bar graph and the red line graph respectively show the gene expression levels of qRT-PCR and transcriptome of the gene in different plant tissues of S and Y, and r is the Spearman correlation coefficient of the expression levels of qRT-PCR and transcriptome of this gene. The correspondence between gene names and function predictions was as follows: LHCB1: TRINITY_DN3700_c0_g3; LHCA2: TRINITY_DN4953_c0_g1, TRINITY_DN8143_c0_g1; LHCB3: TRINITY_DN5179_c0_g1; LHCB5: TRINITY_DN10275_c0_g1; CYP77A: TRINITY_DN18805_c0_g1, TRINITY_DN22380_c0_g1; CYP86A4S: TRINITY_DN2115_c0_g1, TRINITY_DN237_c0_g1; CER1: TRINITY_DN26048_c0_g1; Fatty acyl-CoA reductase, TRINITY_DN3165_c0_g1; GMC oxidoreductase: TRINITY_DN44647_c0_g1; Alcohol-forming fatty acyl-CoA reductase: TRINITY_DN9882_c0_g1; NRT2: TRINITY_DN9053_c0_g1, TRINITY_DN3985_c0_g1; Carbonic anhydrase: TRINITY_DN4039_c0_g1, TRINITY_DN11353_c0_g1; nirA: TRINITY_DN5900_c0_g3.
