# Supplementary figures and images for: Transcriptomic profiling reveals response mechanisms of Lactuca indica seedlings to seawater irrigation stress
Source: Front Plant Sci. 2025 Jun 3;16:1599564. doi: 10.3389/fpls.2025.1599564 (PMC12188455; doi:10.3389/fpls.2025.1599564)

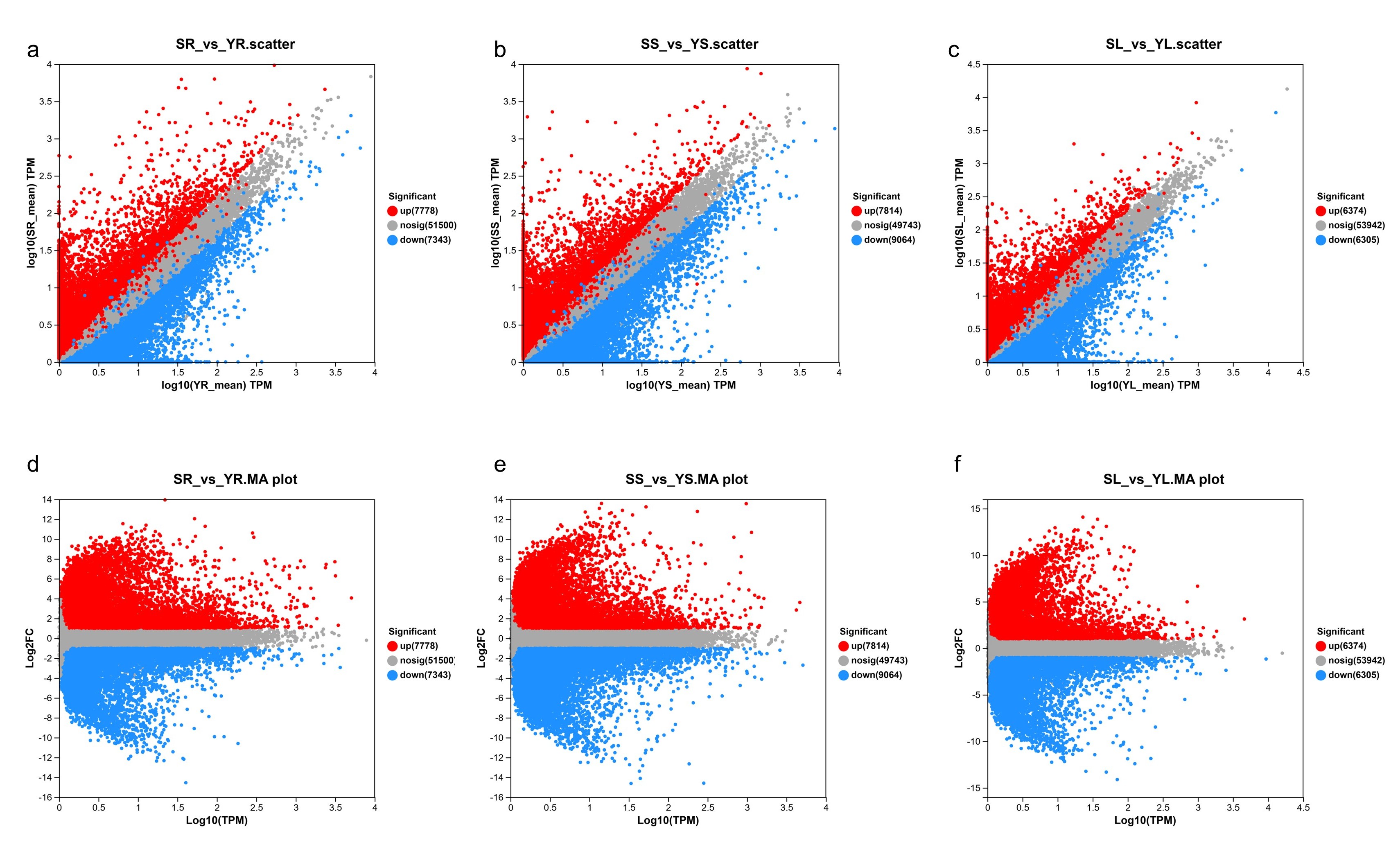

Supplement: Supplementary Figure 1 — Scatter plot of DEGs expression level difference statistics and MA plot. (a-c) Scatter plot of differentially expressed genes in different plant tissue parts of S and Y, for example, in SR vs. YR, the meaning of upregulated expression genes is that the expression level of the gene in SR is relatively higher than that in YR. A-c, the horizontal and vertical coordinates respectively represent SR and YR (a). The expression levels of unigene (the longest transcript in the transcript cluster is regarded as unigene) in each of the three control groups SS and YS (b), SL and YL (c), with two samples in each group. Here, the values on the horizontal and vertical coordinates have been logarithmically processed, and each point represents a specific unigene. In the figure, the red dots represent significantly upregulated unigene, the blue dots represent significantly downregulated unigene, and the gray dots represent unigene with non-significant differences. After mapping all unigene onto it, the closer the point is to 0, the lower the expression level. Those points with a greater degree of deviation from the diagonal indicate that the expression difference of the unigene between the two samples is greater. (d-f) S and Y MA plots of differentially expressed genes in different plant tissue parts, d.SR vs. YR, e. SS vs. YS, and f. SL vs. YL. The horizontal axis represents the calculation based on the expression values of the difference control groups, and the vertical axis represents the expression changes of unigene between the difference control groups obtained from the difference expression analysis. The values of both the horizontal and vertical axes have been logarithmically processed. Each point in the figure represents a specific unigene. By default, red points indicate significantly up-regulated unigene, blue points indicate significantly down-regulated unigene, and gray points indicate non-significantly different unigene. After mapping all the unigene onto it, it can be [file Image1.jpeg]

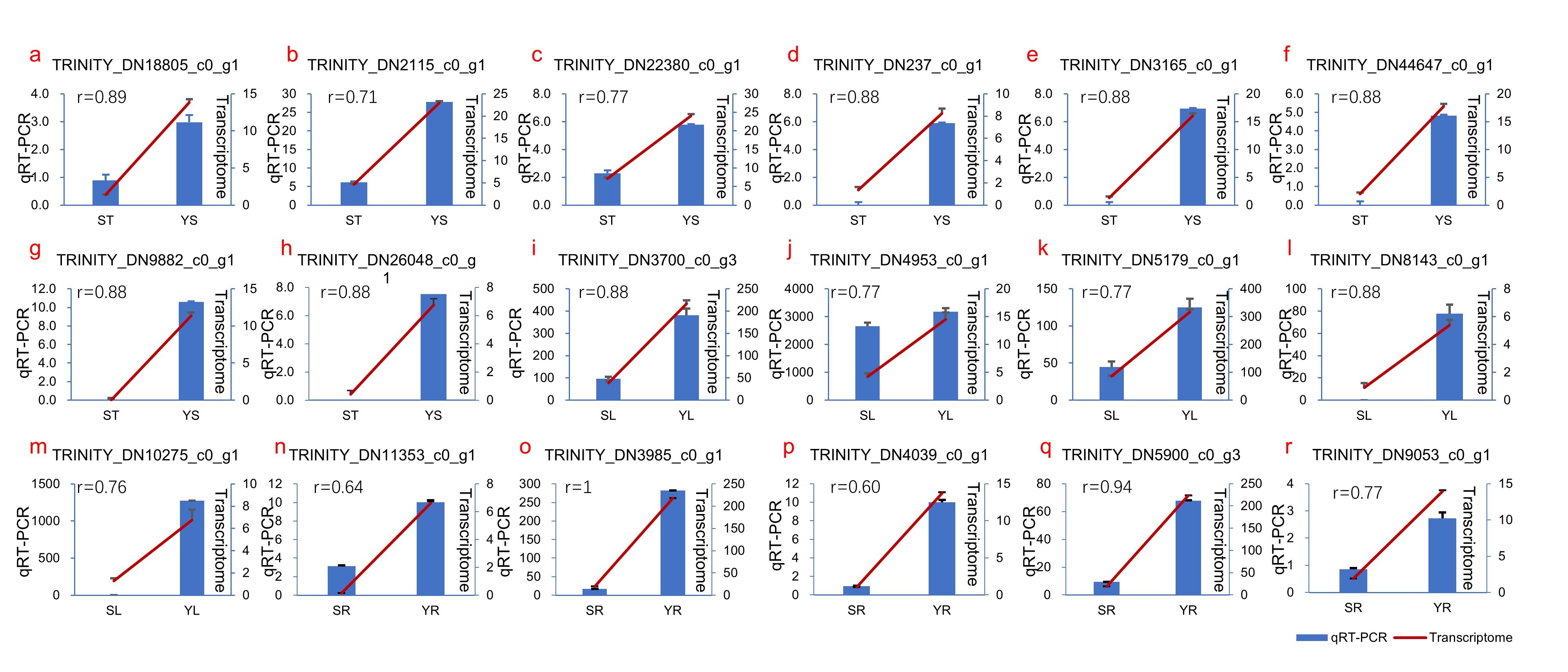

Supplement: Supplementary Figure 2 — Analysis of qRT-PCR results and transcriptome expression levels and their correlations. (a-h) Gene expression in the stem. (i-m) Gene expression in the leaves. (n-r) Gene expression in the root. The header of the table represents the gene id. The blue bar graph and the red line graph respectively show the gene expression levels of qRT-PCR and transcriptome of the gene in different plant tissues of S and Y, and r is the Spearman correlation coefficient of the expression levels of qRT-PCR and transcriptome of this gene. The correspondence between gene names and function predictions was as follows: LHCB1: TRINITY_DN3700_c0_g3; LHCA2: TRINITY_DN4953_c0_g1, TRINITY_DN8143_c0_g1; LHCB3: TRINITY_DN5179_c0_g1; LHCB5: TRINITY_DN10275_c0_g1; CYP77A: TRINITY_DN18805_c0_g1, TRINITY_DN22380_c0_g1; CYP86A4S: TRINITY_DN2115_c0_g1, TRINITY_DN237_c0_g1; CER1: TRINITY_DN26048_c0_g1; Fatty acyl-CoA reductase, TRINITY_DN3165_c0_g1; GMC oxidoreductase: TRINITY_DN44647_c0_g1; Alcohol-forming fatty acyl-CoA reductase: TRINITY_DN9882_c0_g1; NRT2: TRINITY_DN9053_c0_g1, TRINITY_DN3985_c0_g1; Carbonic anhydrase: TRINITY_DN4039_c0_g1, TRINITY_DN11353_c0_g1; nirA: TRINITY_DN5900_c0_g3. [file Image2.jpeg]
